# Effect of Hand Grip Strength on Perioperative Outcomes in Older Female Patients Scheduled for Total Knee Arthroplasty Under General Anesthesia—A Prospective Observational Study

**DOI:** 10.3390/jcm15020463

**Published:** 2026-01-07

**Authors:** Sangho Lee, Doh Yoon Kim, Minsu Kong, Ann Hee You, Jung Eun Kim, Hee Yong Kang

**Affiliations:** 1 Department of Anesthesiology and Pain Medicine, Kyung Hee University College of Medicine, Kyung Hee University Hospital, Seoul 02447, Republic of Korea; sangholee@khu.ac.kr (S.L.); dlmddyk@khu.ac.kr (D.Y.K.); kmsdragonfly@khu.ac.kr (M.K.); ahyou@khu.ac.kr (A.H.Y.); geri200@khu.ac.kr (J.E.K.); 2 Graduate School of Medicine, Kyung Hee University, Seoul 02447, Republic of Korea

**Keywords:** clinical frailty scale, delirium, older female patients, estimated glomerular filtration rate, general anesthesia, hand grip strength, postoperative outcomes, total knee arthroplasty

## Abstract

**Background**: This study aims to evaluate the effect of hand grip strength (HGS) on perioperative outcomes—particularly postoperative delirium (POD)—in patients scheduled for total knee arthroplasty (TKA). **Methods**: Older female patients, aged ≥ 65 years, who were scheduled for TKA under general anesthesia were enrolled in this study. We measured preoperative HGS and clinical frailty scale. The primary outcome was the incidence of POD within 30 days of surgery. Secondary outcomes included intraoperative hypotension, surgical site infection, postoperative pulmonary complications, postoperative nausea and vomiting, acute kidney injury, postoperative urinary retention, and hospital length of stay. **Results**: The final analysis was conducted on 78 participants. The median HGS was 17.9 kg, the patients were divided into Weak (HGS ≤ 17.9, *n* = 39) and Strong groups (HGS > 17.9, *n* = 39). POD was more prevalent in the Weak group (23.1% vs. 0.0%, *p* = 0.005). As secondary outcomes, there were no significant differences between the two groups, except the postoperative estimated glomerular filtration rate (101 [90; 120.5] mL/min/1.73 m^2^ in the Weak group vs. 122 [104; 138] mL/min/1.73 m^2^ in the Strong group; *p* = 0.007). In the receiver operating characteristic curve analysis of POD occurrence according to HGS, the cutoff value was 17.5 (area under curve 0.88, *p* < 0.001). In univariate logistic regression analysis, age and HGS were associated with the occurrence of POD. In multivariate logistic regression analysis, HGS was the only factor that affects POD. For each 1 kg increase in HGS, the risk of POD decreased by 28% (Odds ratio: 0.72). **Conclusions**: In this study, lower preoperative HGS was significantly associated with the occurrence of POD.

## 1. Introduction

Total knee arthroplasty (TKA) is an established and effective surgical treatment for patients with advanced knee osteoarthritis who fail to respond to conservative management [1]. With progressive population aging, the number of older adults undergoing TKA continues to increase worldwide. Although advances in surgical techniques, anesthesia, and perioperative care have improved overall outcomes, older patients remain particularly vulnerable to postoperative complications, which may result in prolonged hospitalization, delayed functional recovery, increased healthcare utilization, and higher medical costs [2,3,4,5]. Consequently, identifying preoperative risk factors that can predict adverse perioperative outcomes is of substantial clinical importance.

Among postoperative complications, postoperative delirium (POD) is one of the most frequent and clinically relevant neurocognitive disorders in older surgical patients [6]. POD is characterized by an acute and fluctuating disturbance of attention and cognition and has been consistently associated with poor short- and long-term outcomes, including prolonged length of stay, impaired rehabilitation, increased risk of institutionalization, long-term cognitive decline, and mortality [7]. In patients undergoing major orthopedic surgery such as TKA, POD not only complicates postoperative management but also interferes with early mobilization and functional recovery, which are essential for optimal surgical outcomes. Given its substantial clinical and socioeconomic impact, POD was prospectively defined as the primary outcome of the present study.

Frailty, a multidimensional syndrome reflecting decreased physiological reserve and increased vulnerability to stressors, has emerged as a key determinant of adverse postoperative outcomes, including POD, in older adults [8]. Various instruments have been proposed to assess frailty, such as the Clinical Frailty Scale (CFS), radiologic measurements of muscle mass, and anthropometric assessments of specific muscle groups or limb circumferences [9,10,11,12,13]. While informative, these methods often require additional examinations, specialized equipment, or substantial time, limiting their feasibility in routine perioperative practice.

Hand grip strength (HGS) has gained increasing attention as a simple, noninvasive, and objective surrogate marker of frailty and overall health status [14]. HGS can be measured within seconds using a handheld dynamometer and has been shown to correlate with sarcopenia, nutritional status, physical function, and long-term outcomes in older populations. Previous studies have demonstrated associations between low HGS and postoperative complications, cognitive impairment, and mortality across various surgical and medical settings [15,16,17]. However, evidence focusing specifically on the relationship between preoperative HGS and POD in patients undergoing TKA remains limited.

In addition, women represent the majority of patients undergoing TKA, and sex-related differences in muscle strength, frailty progression, and postoperative outcomes have been reported [18]. To reduce heterogeneity and enhance the interpretability of the results, this study focused exclusively on older female patients. We also assessed preoperative frailty using both HGS and CFS to better characterize patients’ baseline functional status.

Accordingly, we hypothesized a priori that lower preoperative HGS would be associated with a higher incidence of POD. The primary aim of this prospective observational study was to evaluate the association between preoperative HGS and POD, while secondary objectives included examining the impact of HGS on other perioperative outcomes in older female patients scheduled for TKA under general anesthesia.

## 2. Materials and Methods

### 2.1. Study Design, Ethics, and Registry

This prospective observational study was designed to evaluate the effect of HGS on postoperative outcomes in older female patients scheduled for TKA under general anesthesia. This trial was approved by the Institutional Review Board of Kyung Hee University Hospital (KHUH 2023-03-069) on 28 April 2023 and written informed consent was obtained from all participants in the trial. This study was registered prior to patient enrollment at the Clinical Research Information Service (No. KCT0008488; principal investigator: Hee Yong Kang; registration date: 2 June 2023) and was conducted in accordance with the Declaration of Helsinki. The study protocol is available from the Clinical Research Information Service. This study complied with the Strengthening the Reporting of Observational Studies in Epidemiology (STROBE) checklist.

### 2.2. Participants

Older female patients age ≥ 65 years who were scheduled for elective primary TKA under general anesthesia at single tertiary medical center were eligible for inclusion. The exclusion criteria included planned revision or bilateral TKA, inability to measure HGS, American Society of Anesthesiologists physical status class ≥ III, history of kidney disease, cerebrovascular disease, altered mental status, and a body mass index > 35 kg/m^2^. Recruitment began in June 2023 and ended in October 2023.

### 2.3. Outcomes

The primary outcome was the incidence of POD within 30 days after surgery, defined a priori. POD was defined as acute change in the patient’s mental status and a diagnosis of delirium by a psychiatrist using the Confusion Assessment Method [19,20]. The psychiatrist was blinded to all preoperative data, including HGS.

Secondary outcomes included intraoperative hypotension, surgical site infection (SSI), postoperative pulmonary complications (PPC), postoperative nausea and vomiting (PONV), acute kidney injury (AKI), postoperative urinary retention (POUR) until postoperative day 30, and postoperative hospital length of stay. Intraoperative hypotension was defined as a ≥20% reduction in systolic blood pressure from baseline [21]. SSI was defined as postoperative antibiotic administration or wound revision due to an abscess formation [22,23]. PPC was defined according to the European Joint Task Force published guidelines for perioperative clinical outcome. These include respiratory infection, respiratory failure, pleural effusion, atelectasis, pneumothorax, bronchospasm, and aspiration pneumonitis [24]. PONV was defined as the patient complained of nausea or vomiting, or antiemetics were administered [25]. AKI was evaluated using the Kidney Disease: Improving Global Outcomes criteria and was defined as an increase in serum creatinine (Cr) level ≥ 0.3 mg/dL from baseline [26]. POUR was defined as urinary retention ≥ 300 mL on bladder scan or if a urinary catheterization was performed while the patient complained of suprapubic discomfort [27,28].

Postoperative complications were assessed for up to 30 days after surgery to ensure comprehensive evaluation of patient safety, consistent with commonly used postoperative follow-up periods in clinical outcome studies [29]. Postoperative outcomes were assessed by a single researcher who was blinded to the patients’ preoperative data, including HGS, based on medical records up to 30 days after surgery.

### 2.4. Procedures

After entering the operating room, HGS and CFS were measured. The HGS was measured once on each hand using a manometer (Camry digital hand dynamometer; Camry scale, South El Monte, CA, USA), and the stronger value was used in the data analysis. The CFS was assessed using the Canadian Study of Health and Aging Clinical Frailty Scale, which categorizes frailty into nine levels by a single researcher (Supplementary Table S1) [9,30]. Standard patient monitoring was performed according to clinical practice guidelines. Immediately before anesthesia induction, glycopyrrolate 0.2 mg was administered as an anticholinergic agent, and palonosetron 0.075 mg was given as an antiemetic. For anesthesia induction, propofol at 1–1.5 mg/kg and rocuronium 0.8 mg/kg were administered. To minimize the impact of deep sedation on the occurrence of POD, anesthesia was maintained with sevoflurane at 0.8–1.2 minimum alveolar concentration, targeting a bispectral index 40–60 [31]. Remifentanil was administered at 0.05–0.2 mcg/kg/min while monitoring blood pressure and heart rate. When intraoperative hypotension occurred, 50 mcg of phenylephrine or 5 mg of ephedrine was administered depending on the vital signs. At the end of the surgery, 2 mg/kg of sugammadex was administered, and extubation was performed after confirming the patient’s spontaneous ventilation. Fentanyl-based (15 mcg/kg) IV patient-controlled analgesia was administered until postoperative day 2.

### 2.5. Sample Size Calculation

The authors conducted a pilot study on the incidence of delirium according to HGS who underwent TKA for sample size calculation. This study was conducted on 36 patients, and median HGS was 17.4 kg [12.3; 22.7]. Delirium occurred in four patients in the group with HGS lower than median value (incidence of POD: 0% (0/18) in strong group vs. 22.2% (4/18) in weak group). Based on these data, as a result of G-power analysis (α error 0.05, power 0.8), the target number of participants was 70. Considering the dropout rate of 10%, the calculated sample size was 78.

### 2.6. Statistical Analysis

The study data were presented as medians [interquartile ranges] or numbers (%), as appropriate. The Shapiro–Wilk test was performed to evaluate the normality of continuous variables. Independent variable *t*-tests or Wilcoxon rank–sum tests were used to analyze normal or non-normal distributed continuous variables, respectively. Chi-square test or Fisher’s exact test was performed to analyze categorical variables. The optimal HGS associated with POD was assessed based on clinical relevance and the Youden index (sensitivity (%) + specificity (%) − 100) from the Receiver operating characteristic (ROC) curve analysis. Univariate logistic regression analysis was performed to evaluate the factors causing POD, and all variables with *p* < 0.2 or previously described clinically important factors were included in the multivariate analysis. Covariates were adjusted for age, diabetes, hypertension, hematocrit, alanine transaminase (ALT), blood urea nitrogen, estimated glomerular filtration rate (eGFR), CFS, HGS, intraoperative hypotension and anesthesia time. Multicollinearity among the variables was evaluated via a variance inflation factor (VIF), with the VIF < 10 indicating no multicollinearity. Model calibration was assessed using the Hosmer–Lemeshow goodness-of-fit test. A post hoc power analysis was performed based on the data obtained in the current study. Statistical analysis were performed using the commercial statistical SPSS software (version 22.0; IBM Corp., Armonk, NY, USA). Differences were considered statistically significant at two-tailed *p* values < 0.05.

## 3. Results

### 3.1. Study Participants and Patient Characteristics

**Figure 1:**
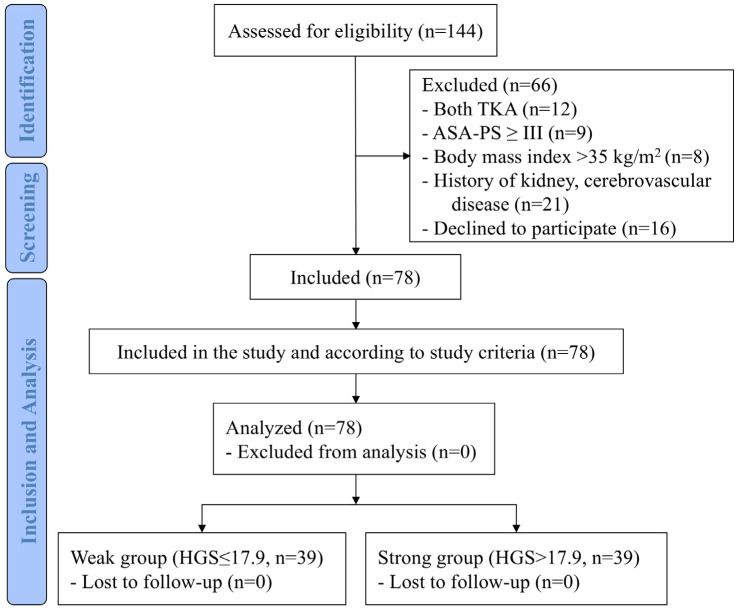
Patient flow chart. ASA-PS, American Society of Anesthesiologists physical status; HGS, hand grip strength; TKA, total knee arthroplasty.

We screened 144 patients, of whom 66 were excluded, and 78 were enrolled. The median HGS value for all participants was 17.9 kg [12.6; 21.8], 39 patients were equally divided into Weak (HGS ≤ 17.9) or Strong (HGS > 17.9) groups. Finally, 78 patients were analyzed without follow-up loss (Figure 1).

In demographic data of the Weak group, the age (75 [72; 80] vs. 71 [68; 75], *p* = 0.006) was significantly higher and ALT (17 [14; 21] vs. 21 [17; 24], *p* = 0.019) were significantly lower than the Strong group. The HGS were 12.6 [10.4; 15.9] and 21.8 [20.1; 24.1] in the Weak and Strong group, respectively. Other patient characteristics were comparable in both groups (Table 1).

### 3.2. Primary Outcome

In the perioperative outcome evaluation, POD occurred more frequently in the Weak group than the Strong group (9 (23.1%) vs. 0 (0.0%), *p* = 0.005) (Table 2).

**Figure 2:**
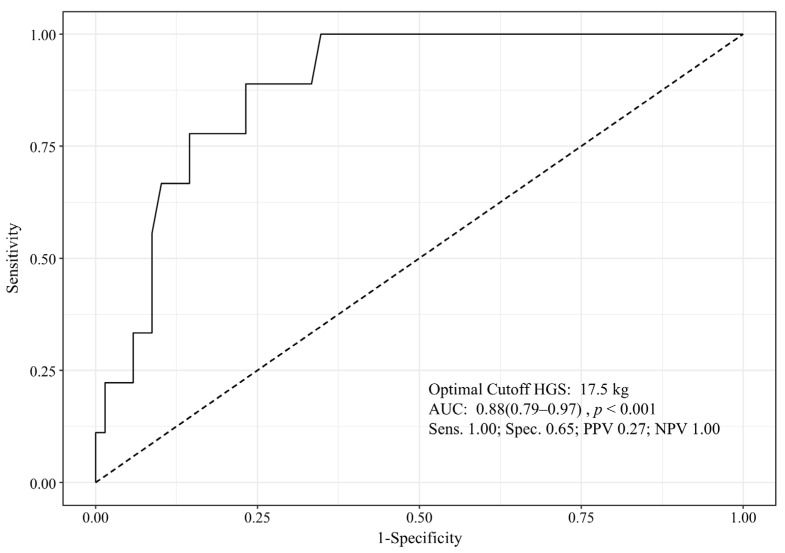
Receiver operating characteristic curve analysis according to postoperative delirium occurrence among all patients of preoperative hand grip strength. AUC, area under curve; HGS, hand grip strength; NPV, negative predictive value; PPV, positive predictive value; Sens., sensitivity; Spec., specificity.

In the ROC curve analysis for the occurrence of POD according to HGS, the optimal cutoff value was 17.5 kg (Figure 2).

### 3.3. Secondary Outcomes

When divided into two groups based on HGS median 17.9, other postoperative outcomes were comparable in both groups except eGFR. The postoperative eGFR was significantly lower in the Weak group (101 [90; 121] vs. 122 [104; 138], *p* = 0.007) (Table 2).

In univariate logistic regression analysis, age, and HGS were found to be associated with the occurrence of POD. In multivariate logistic regression analysis, only HGS was associated with the occurrence of POD. For each 1 kg increase in HGS, the risk of POD decreased by 28% (Odds ratio: 0.72) (Table 3; Supplementary Figure S1). According to the estimated VIFs (<10), multicollinearity had a minimal impact on the results (Table 3). The Hosmer–Lemeshow test indicated good model calibration (χ^2^ = 3.859, df = 8, *p* = 0.870). Overall model performance was further supported by a low residual deviance relative to degrees of freedom (32.9/66 = 0.50) and a high explanatory power, as reflected by the Nagelkerke pseudo-R^2^ of 0.498.

During the study period, no serious complications, including mortality, occurred, and all participants were discharged to their home.

### 3.4. Post Hoc Power Analysis

Based on the data obtained in the current study, a post hoc power analysis was performed to determine the effect of the HGS on the occurrence of POD. Using the POD incidence of the Weak group and Strong group being 23.1% (9/39) and 0% (0/39), respectively, with an α error of 0.05, the power of this study was calculated to be 0.91.

## 4. Discussion

In this study, we found that lower preoperative HGS was associated with an increased incidence of POD in older female patients scheduled for TKA under general anesthesia. There have been reports suggesting that HGS can be used to assess patients’ overall health status and frailty [32]. Frailty is known to be a risk factor for POD [33]. Therefore, the higher incidence of POD observed in patients with low HGS is likely attributable to their increased frailty. The incidence of POD has been reported to vary widely, ranging from approximately 10–50%. It is considered a major factor contributing to prolonged hospital length of stays and increased healthcare costs [34,35]. By utilizing the findings of this study to predict and prepare for POD in patients with low preoperative HGS, limited healthcare resources can be allocated more efficiently. Measurement of HGS can be easily incorporated into routine preoperative assessments, allowing for early identification of high-risk patients without additional cost or time burden. For patients with low preoperative HGS, targeted interventions such as preoperative nutritional counseling, resistance-based prehabilitation programs aimed at improving muscle mass and strength, and closer perioperative monitoring for cognitive changes may help achieve better postoperative outcomes [36,37]. In addition, these patients may benefit from tailored anesthetic and analgesic strategies, early mobilization, and enhanced delirium prevention protocols, including optimized pain control, sleep promotion, and minimization of deliriogenic medications. Collectively, incorporating HGS into perioperative risk stratification may facilitate individualized care pathways and contribute to improved patient safety and recovery.

The association between HGS and the occurrence of POD has been mentioned in numerous previous studies [32,38,39]. In addition to previous studies, this study conducted a prospective analysis focusing solely on a specific patient population, allowing for a clearer determination of HGS. In the study by Qian et al. the cutoff value for HGS in female was reported as 18.05 kg [38]. Similarly, the Revised Japanese version of the Cardiovascular Health Study criteria [40] suggests a reference value of 18 kg for female. These values are comparable to the median HGS of 17.9 kg in this study, supporting the reliability of our data.

In this study, the optimal cutoff value determined by ROC curve analysis was 17.5 kg. This value is similar to both the median value of our study population and the reference values reported in previous studies [38,40]. However, its low positive predictive value limits its utility as a standalone screening tool in clinical practice. Therefore, if a patient presents with low HGS, it is important to consider this result in conjunction with other clinical findings and test results to anticipate and prepare for the possibility of POD. On the other hand, the high negative predictive value suggests that patients with higher HGS are less likely to develop POD, which could help avoid unnecessary use of additional medical resources.

In this study, CFS was measured alongside HGS preoperatively. Numerous studies have reported associations between CFS and HGS, as well as between CFS and POD [41,42,43]. However, this study did not find a statistically significant association related to CFS. This may be attributed to the subjective nature of CFS assessment. Since CFS is evaluated through a clinician observing and interacting with the patient, the assessor’s subjective judgment can influence the results [44]. Although we attempted to minimize bias by having a single researcher assess CFS, no significant findings were obtained. In contrast, HGS provides a numerical representation of a patient’s condition, offering the advantage of greater objectivity regardless of the researcher.

In the demographic data, the Weak group was older. This is an expected finding, as aging is associated with decreased HGS, a relationship that has been well-documented in numerous studies [45,46]. In the univariate logistic regression analysis, age was found to be significantly associated with POD. However, in the multivariate logistic regression analysis, only HGS remained a significant factor. The association between age and POD has been reported in previous studies [47,48]. The current study focused on patients aged ≥ 65, which may have led to an underestimation of the risk of delirium associated with aging. Our findings suggest that in older patients aged ≥ 65, HGS may be more strongly associated with POD than age itself. Future studies should investigate age-adjusted HGS thresholds to better distinguish age-related physiological decline from true frailty and to minimize potential confounding effects. Additionally, the weak group demonstrated statistically lower preoperative ALT levels and postoperative eGFR. ALT reflects hepatocellular function and may influence the hepatic metabolism of anesthetic agents such as propofol and remifentanil. Therefore, lower ALT levels could theoretically be associated with altered drug metabolism and might indirectly contribute to the pathogenesis of POD through liver–brain interactions. However, as both ALT and postoperative eGFR values were largely within the normal range in both groups, these differences are unlikely to have significant clinical implications. Further investigation is warranted in future studies involving patients with overt hepatic dysfunction.

Accumulating evidence indicates that systemic inflammation and neuroinflammation play central roles in the pathogenesis of POD [49]. Surgical trauma and anesthesia trigger a systemic immune response, leading to the release of pro-inflammatory cytokines such as interleukin-6 (IL-6), C-reactive protein (CRP), interleukin-8, and interleukin-1β into the circulation. These inflammatory mediators can disrupt the integrity of the blood–brain barrier, activate microglia and astrocytes, and promote central nervous system inflammation, which may impair neuronal function and contribute to delirium onset. Meta-analytic data have demonstrated that patients who develop POD exhibit significantly elevated levels of inflammatory biomarkers, including IL-6, CRP, and other cytokines, compared with those who do not develop delirium, supporting the concept that neuroinflammation is a key mechanism underlying POD development [50]. In the current study, preoperative white blood cell (WBC) counts were measured; however, there were limitations in assessing additional inflammatory biomarkers solely for research purposes. Preoperative WBC levels did not differ significantly between the two groups and were not associated with POD in univariate logistic regression analysis. As only relatively healthy patients (American Society of Anesthesiologists physical status I–II) were included, the baseline inflammatory burden may have been insufficient to exert a measurable influence on postoperative outcomes. Future studies investigating POD should incorporate a more comprehensive evaluation of inflammatory markers to better elucidate their role in POD pathophysiology.

A key strength of this study is the specific focus on older female patients, which helped minimize biases related to patient characteristics in postoperative outcomes. Additionally, surgery was performed by a single surgeon, minimizing variability in surgical techniques, and data were collected by a single researcher to reduce measurement biases caused by inter-researcher differences. To ensure the fairness of the results, the researcher assessing postoperative outcomes was blinded to the patients’ preoperative information. Lastly, both HGS and CFS were measured to gain a more comprehensive understanding of patients’ preoperative conditions. This allowed us to conclude that in older patients, HGS is more closely associated with POD than CFS.

This study has several limitations. First, the method for measuring HGS has not been clearly standardized. In this study, HGS was measured once on each hand, and the higher value was used for analysis. A previous study suggested measuring HGS three times on each hand [51], but we observed a tendency for HGS to decrease with repeated measurements. Although repeated measurements may improve reproducibility, single measurements per hand were chosen to minimize patient fatigue and improve feasibility in a real-world clinical setting. Second, we did not assess patients’ Mini-Mental State Exam (MMSE) scores preoperatively or postoperatively. Numerous previous studies have reported an association between cognitive function and HGS [52,53]. However, since this study aimed to evaluate various postoperative outcomes, we omitted MMSE assessments, which require considerable time and effort. Third, this study was conducted exclusively in older female patients undergoing TKA, and the relatively small sample size may limit the generalizability of the findings to a broader population. Although restricting the study population to female patients limits external validity, this approach allowed for a more homogeneous cohort and reduced potential confounding effects related to sex-specific differences in muscle strength and frailty. Moreover, the prospective design and the clearly defined primary outcome strengthen the internal validity of this study. This investigation was not intended to be a population-based analysis reflecting the overall incidence of TKA, but rather a hypothesis-driven prospective study focusing on a clinically relevant high-risk subgroup. Future large-scale prospective studies, including male patients, very elderly patients (>80 years), younger patients, and individuals undergoing TKA under regional anesthesia, are warranted to validate and generalize these findings. Fourth, postoperative pain was not evaluated. However, IV patient-controlled analgesia was administered to all patients to minimize postoperative pain. Fifth, complications treated at other hospitals within 30 days after surgery could not be verified. However, most patients returned to our institution for continuous follow-up after surgery. Sixth, we did not measure parameters commonly used to assess frailty in clinical practice, such as psoas muscle size or lower limb circumference [54,55]. However, these measurements require additional tests and time. The authors selected HGS and the CFS because both can be assessed quickly and easily in routine clinical practice without requiring additional testing, prolonged assessment time, or extra costs, thereby supporting the efficient utilization of limited healthcare resources. Moreover, the use of HGS in this study yielded clinically meaningful results. The present study focused on pragmatic frailty measures with high clinical applicability rather than exhaustive frailty phenotyping. Future studies should compare the impact of these parameters on postoperative outcomes. Lastly, follow-up was limited to 30 days postoperatively, preventing the assessment of long-term postoperative outcomes such as mortality. However, no mortality cases were observed during the study period.

## 5. Conclusions

In conclusion, lower preoperative HGS was associated with the occurrence of POD. For each 1 kg increase in HGS, the risk of POD decreased by 28% (Odds ratio: 0.72). By applying these findings to clinical practice, we aim to promote the efficient use of medical resources and support the rapid postoperative recovery of patients.

## Figures and Tables

**Table 1:** Patient characteristics according to weak or strong hand grip strength.

Variable	Total (*n* = 78)	Weak Group (HGS ≤ 17.9) (*n* = 39)	Strong Group (HGS > 17.9) (*n* = 39)	*p*-Value
Age, year	74 [69; 78]	75 [72; 80]	71 [68; 75]	0.006 *
BMI, kg/m^2^	25.6 [23.6; 27.4]	25.3 [22.8; 27.2]	26.0 [23.9; 27.6]	0.363
ASA-PS class I/II, *n* (%)	24 (30.8%)/ 54 (69.2%)	10 (25.6%)/ 29 (74.4%)	14 (35.9%)/ 25 (64.1%)	0.462
Diabetes, *n* (%)	26 (33.3%)	12 (30.8%)	14 (35.9%)	0.810
HTN, *n* (%)	48 (61.5%)	26 (66.7%)	22 (56.4%)	0.485
WBC, ×10^3^/μL	5.87 [4.82; 7.01]	5.79 [4.80; 7.37]	5.94 [5.02; 6.69]	0.988
Hematocrit, %	39.0 [36.3; 40.9]	38.7 [35.9; 40.2]	39.3 [37.5; 41.1]	0.083
AST, U/L	25 [22; 28]	23 [22; 27]	25 [21; 30]	0.309
ALT, U/L	20 [14; 23]	17 [14; 21]	21 [17; 24]	0.019 *
BUN, mg/dL	16 [14; 19]	17 [14; 19]	16 [14; 19]	0.591
eGFR, mL/m/1.73 m^2^	98 [86; 112]	96 [85; 112]	104 [91; 113]	0.183
ESR, mm/h	23 [15; 31]	23 [10; 30]	23 [16; 33]	0.289
CFS	4 [3; 5]	4 [3; 5]	4 [3; 4]	0.084
HGS, kg	17.9 [12.6; 21.8]	12.6 [10.4; 15.9]	21.8 [20.1; 24.1]	<0.001 *

Continuous data are presented as median [interquartile ranges] and categorical data as *n* (%). * Statistical significance between groups. ALT, alanine transaminase; ASA-PS, American Society of Anesthesiologists physical status; AST, aspartate transaminase; BMI, body mass index; BUN, blood urea nitrogen; CFS, clinical frailty scale; eGFR, estimated glomerular filtration rate; ESR, erythrocyte sedimentation rate; HGS, hand grip strength; HTN, hypertension; WBC, white blood cell.

**Table 2:** Perioperative data and outcomes until postoperative days 30.

Variable	Total (*n* = 78)	Weak Group (HGS ≤ 17.9) (*n* = 39)	Strong Group (HGS > 17.9) (*n* = 39)	*p*-Value
Intraoperative hypotension, *n* (%)	44 (56.4%)	19 (48.7%)	25 (64.1%)	0.254
Anesthesia time, min	140 [130; 150]	140 [130; 150]	140 [130; 150]	0.836
POD, *n* (%)	9 (11.5%)	9 (23.1%)	0 (0.0%)	0.005 *
SSI, *n* (%)	2 (2.6%)	1 (2.6%)	1 (2.6%)	1.000
PPC, *n* (%)	5 (6.4%)	4 (10.3%)	1 (2.6%)	0.355
PONV, *n* (%)	32 (41.6%)	19 (48.7%)	13 (34.2%)	0.289
AKI, *n* (%)	1 (1.3%)	1 (2.6%)	0 (0.0%)	1.000
Postoperative eGFR, mL/m/1.73 m^2^	111 [95; 133]	101 [90; 121]	122 [104; 138]	0.007 *
POUR, *n* (%)	49 (62.8%)	25 (64.1%)	24 (61.5%)	1.000
Hospital LOS, day	6 [6; 7]	6 [6; 7]	6 [6; 7]	0.597

Continuous data are presented as median [interquartile ranges] and categorical data as *n* (%). * Statistical significance between groups. AKI, acute kidney injury; eGFR, estimated glomerular filtration rate; LOS, length of stay; POD, postoperative delirium; PONV, postoperative nausea and vomiting; POUR, postoperative urinary retention; PPC, postoperative pulmonary complications; SSI, surgical site infection.

**Table 3:** Univariate and multivariate logistic regression analyses of factors associated with postoperative delirium.

	Univariable		Multivariable		
	OR (95% CI)	*p*-Value	OR (95% CI)	*p*-Value	VIF ^†^
Age	1.24 (1.08, 1.47)	0.005 *	0.96 (0.74, 1.21)	0.750	2.236
BMI	0.88 (0.67, 1.11)	0.313			
ASA-PS II	1.64 (0.36, 11.60)	0.558			
Diabetes	1.00 (0.20, 4.16)	1.000	2.08 (0.18, 29.75)	0.548	1.731
Hypertension	5.80 (0.99, 110.64)	0.106	3.46 (0.31, 92.84)	0.357	1.276
WBC	1.00 (1.00, 1.00)	0.851			
Hematocrit	0.85 (0.67, 1.06)	0.145	0.94 (0.64, 1.35)	0.753	1.628
AST	0.95 (0.83, 1.03)	0.426			
ALT	0.89 (0.76, 0.99)	0.079	0.91 (0.73, 1.07)	0.363	1.468
BUN	1.09 (0.96, 1.24)	0.168	1.03 (0.81, 1.31)	0.786	1.696
eGFR	0.99 (0.95, 1.02)	0.354	1.01 (0.96, 1.07)	0.698	1.595
ESR	1.00 (0.95, 1.04)	0.991			
CFS	1.56 (0.96, 2.62)	0.075	1.37 (0.67, 3.17)	0.409	1.895
HGS	0.71 (0.55, 0.86)	0.002 *	0.72 (0.48, 0.93)	0.038 *	2.396
Intraoperative hypotension	1.63 (0.40, 8.23)	0.512	2.60 (0.37, 21.93)	0.341	1.232
Anesthesia time	1.02 (0.97, 1.06)	0.491	1.06 (0.98, 1.15)	0.167	1.934

* Statistical significance. ^†^ The VIF < 10 indicated no multicollinearity. ALT, alanine aminotransferase; ASA-PS, American Society of Anesthesiologists physical status; AST, aspartate aminotransferase; BMI, body mass index; BUN, blood urea nitrogen; CFS, clinical frailty scale; CI, confidence interval; eGFR, estimated glomerular filtration rate; ESR, erythrocyte sedimentation rate; HGS, hand grip strength; OR, odds ratio; VIF, variance inflation factor; WBC, white blood cell.

## Data Availability

The data supporting the findings of this study are available from the corresponding author upon reasonable request. Public access to the dataset is restricted due to privacy and ethical constraints.

## Supplementary Materials

The following supporting information can be downloaded at: https://www.mdpi.com/article/10.3390/jcm15020463/s1, Figure S1: Forest plot of the odds ratios for the occurrence of postoperative delirium; Table S1: Canadian study of health and aging clinical frailty scale [10].

## Abbreviations

The following abbreviations are used in this manuscript:

AKI	Acute kidney injury
CFS	Clinical frailty scale
eGFR	Estimated glomerular filtration rate
HGS	Hand grip strength
LOS	Length of stay
POD	Postoperative delirium
PONV	Postoperative nausea and vomiting
POUR	Postoperative urinary retention
PPC	Postoperative pulmonary complications
SSI	Surgical site infection
TKA	Total knee arthroplasty
VIF	Variance inflation factor
