# Supplementary material for: Effect of Hand Grip Strength on Perioperative Outcomes in Older Female Patients Scheduled for Total Knee Arthroplasty Under General Anesthesia—A Prospective Observational Study
Source: J Clin Med. 2026 Jan 7;15(2):463. doi: 10.3390/jcm15020463 (PMC12842274; doi:10.3390/jcm15020463)
Supplement: Supplementary file 1 [file jcm-15-00463-s001.zip › Supplementary Figure S1.pdf]

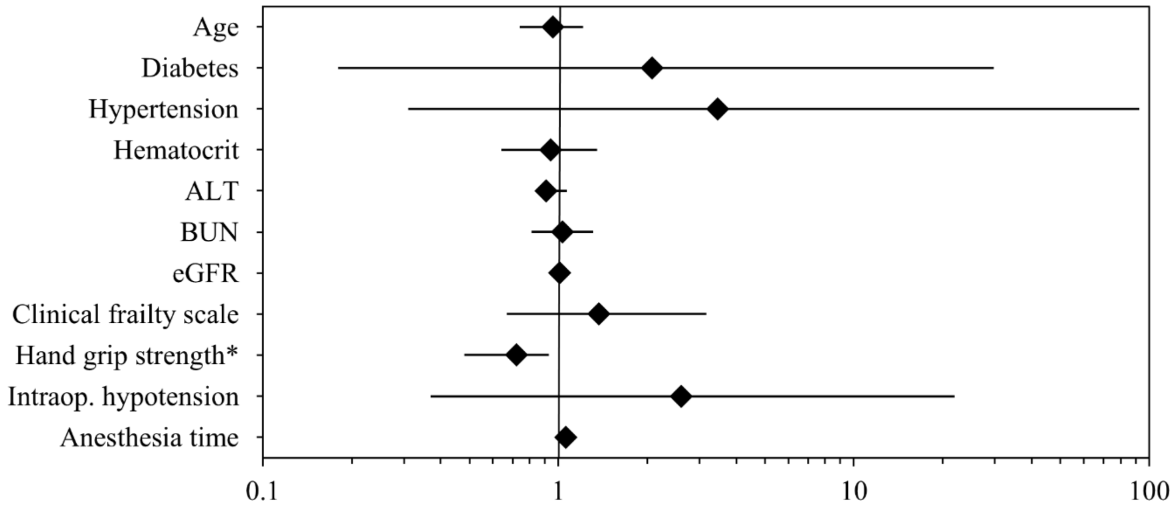

Supplementary figure 1. Forest plot of the odds ratios for the occurrence of postoperative delirium. * Statistical significance. ALT, alanine aminotransferase; BUN, Blood urea nitrogen; eGFR, estimated glomerular filtration rate; Intraop., intraoperative.
