# Supplementary material for: Effect of Hand Grip Strength on Perioperative Outcomes in Older Female Patients Scheduled for Total Knee Arthroplasty Under General Anesthesia—A Prospective Observational Study
Source: J Clin Med. 2026 Jan 7;15(2):463. doi: 10.3390/jcm15020463 (PMC12842274; doi:10.3390/jcm15020463)
Supplement: Supplementary file 1 [file jcm-15-00463-s001.zip › Supplementary Table S1.pdf]

Supplementary table 1. Canadian study of health and aging clinical frailty scale [10]

Scale		Contents
1	**Very Fit**	People who are robust, active, energetic, and motivated. They exercise regularly and are among the fittest for their age.
2	**Well**	No active disease symptoms but less fit than Category 1. They occasionally exercise or are very active occasionally.
3	**Managing Well**	Medical problems exist, but they are well controlled. The person remains independent in daily life.
4	**Vulnerable**	Not dependent on others for daily help but symptoms limit activities. A common complaint is being "slowed up" or feeling tired.
5	**Mildly Frail**	More evident slowing, and they often need help with higher-level activities (e.g., finances, shopping, transportation).
6	**Moderately Frail**	Need help with basic daily activities (e.g., bathing, dressing). Without help, they would have difficulty managing at home.
7	**Severely Frail**	Completely dependent for personal care. They may be stable and not at high risk of dying within 6 months.
8	**Very Severely Frail**	Completely dependent, approaching end of life. They typically cannot recover from even minor illnesses.
9	**Terminally Ill**	Approaching the end of life. A life expectancy of less than 6 months, though they are not otherwise frail.
